# Incremental stability of delayed neural fields: a unifying framework for endogenous and exogenous sources of pathological oscillations

**DOI:** 10.1186/1471-2202-16-S1-P24

**Published:** 2015-12-18

**Authors:** Georgios Is. Detorakis, Antoine Chaillet

**Affiliations:** 1 University Paris Sud, Orsay, Paris, 91400, France; 2 LSS, Supélec, Gif sur Yvette, Paris, 91190, France

## 

Neural fields are integro-differential equations that have been extensively used to model spatiotemporal evolution of neocortical areas (see [1] for a detailed review). Time-delayed neural fields have also been a matter of investigation since they take into account axonal delays [2]. On the other hand, time-delay finite dimensional systems have been used in models of Parkinson's disease: delays have been shown to play a possible role in the generation of pathological neural oscillations linked to motor symptoms of Parkinson disease in a firing-rate model of basal ganglia [3,4]. Nonetheless, these models fail at rendering the spatial distribution of the neural activity of the populations involved. Two possible mechanisms for the onset of pathological oscillations in basal ganglia have been investigated in the literature. The first one, the "endogenous" mechanism, hypothesizes that dopamine depletion tends to increase the synaptic gains between the excitatory neurons of the subthalamic nucleus (STN) and the inhibitory neurons of the external segment of globus pallidus (GPe), thus generating an instability that translates into sustained oscillations. The second one, the "exogenous" mechanism, explains these oscillations onset by a diffusion of spontaneous oscillations from external structures (such as Striatum) to the GPe-STN network [5].

The main goal of this work is to deepen this analysis by providing theoretical conditions under which a network of time-delayed neural field equations is incrementally stable. We believe that incremental stability constitutes an instrumental framework to investigate both the mechanisms evoked above. Indeed, by considering constant inputs to the basal ganglia, incremental stability ensures convergence to a unique equilibrium configuration, thus ruling out the possibility of "endogenous" mechanism for oscillations onset. On the other hand, incremental stability guarantees entrainability to periodic inputs (meaning convergence to a T-periodic solution in response to any T-periodic input), and can thus be useful to unravel the mechanism of pathological diffusion from external structures in the "exogenous" scenario.

Relying on the Razumikhin-Lyapunov approach here we derive these sufficient conditions for incremental stability of delayed neural fields. This theoretical framework thus complements the Krasovskii-Lyapunov approach already used in the literature to address the stability of delayed neural fields equations [6]. Simulations confirm our theoretical expectations and demonstrate that interconnected neural fields can exhibit sustained oscillations, according to either the "endogenous" or the "exogenous" mechanism, depending on the strength of the synaptic weights between the excitatory (STN) and the inhibitory (GPe) populations. The derived theoretical results thus seem to constitute a fertile ground for further investigations based on experimental data, to discriminate between the "endogenous" and the "exogenous" hypotheses for Parkinsonian sustained oscillations in the STN-GPe network.
